# COMPLICATIONS AFTER SURGICAL TREATMENT OF JUPITER’S MONTEGGIA TYPE II FRACTURES

**DOI:** 10.1590/1413-785220233103e267308

**Published:** 2023-07-17

**Authors:** EMANUELLY RIBEIRO GUERRA, LUIZ ROBERTO SOARES DE ARAÚJO FILHO, FERNANDO KENJI KIKUTA, DANIEL ROMANO ZOGBI, GUILHERME GRISI MOURARIA, MAURICIO ETCHEBEHERE

**Affiliations:** 1 Universidade Estadual de Campinas, Faculdade de Ciências Médicas, Departamento de Ortopedia, Reumatologia e Traumatologia, Campinas, SP, Brazil.; 2 Universidade Estadual de Campinas, Faculdade de Ciências Médicas, Departamento de Ortopedia, Reumatologia e Traumatologia, Grupo de Ombro e Cotovelo, Campinas, SP, Brazil.

**Keywords:** Monteggia’s Fracture, Postoperative Complications, Radial Head and Neck Fractures, Fratura de Monteggia, Complicações Pós-Operatórias, Fraturas da Cabeça e do Colo do Rádio

## Abstract

Monteggia fracture-dislocations are rare injuries, affecting about 2-5% of the population. Jesse Jupiter subdivided Bado’s Type II fractures into four types, all of which presented an associated radial head fracture. Associated chondral and ligament injuries can evolve with postoperative complications.

**Objective:**

To evaluate the incidence of complications and risk factors that may influence the postoperative outcomes of Jupiter lesions.

**Methods:**

This retrospective study was conducted with surgically treated patients. The characteristics related to fractures and surgical approaches were evaluated and these variables were correlated with radiographic and functional postoperative complications.

**Results:**

A total of 15 patients were evaluated, mostly men and with a higher prevalence of Types IIA and IID. The most frequent complications were heterotopic ossification and osteolysis around the radial head prosthesis. Postoperative instability occurred only in the lateral collateral ligament. According to MEPS functional score, 53% of the patients evolved with unfavorable outcomes.

**Conclusion:**

The studied cases evolved with high rates of postoperative complications, mainly in Jupiter’s Type IID fractures and associated coronoid fractures. ***Level of Evidence III, Therapeutic Study.***

## RESUMO

A fratura-luxação de Monteggia é uma lesão rara que acomete cerca de 2-5% da população. Jesse Jupiter subdividiu as fraturas tipo II de Bado em quatro tipos, todos eles associados à fratura da cabeça do rádio. As lesões condral e ligamentares associadas podem evoluir com complicações pós-operatórias.

**Objetivo:**

Avaliar a incidência das complicações e os fatores de risco que podem influenciar os resultados pós-operatórios nas fraturas de Monteggia tipo II de Jupiter.

**Métodos:**

Estudo retrospectivo com pacientes tratados cirurgicamente. Avaliaram-se as características relacionadas às fraturas e as técnicas cirúrgicas utilizadas e, em seguida, tais variáveis foram correlacionadas com complicações pós-operatórias radiográficas e funcionais.

**Resultados:**

Foram avaliados 15 pacientes, havendo predomínio do sexo masculino e maior prevalência das fraturas tipo IIA e IID. As complicações mais frequentes foram a ossificação heterotópica e a osteólise ao redor da prótese da cabeça do rádio. A instabilidade pós-operatória ocorreu somente no complexo ligamentar lateral. Funcionalmente, segundo o Mayo Elbow Performance Score, 53% dos pacientes evoluíram com resultados desfavoráveis.

**Conclusão:**

Observou-se alta taxa de complicação pós-operatória, principalmente nas fraturas tipo II-D de Jupiter e naquelas com fratura do coronoide associada. **Nível de Evidência III, Estudo Terapêutico.**

## INTRODUCTION

Monteggia fracture-dislocation is defined as a ulnar fracture associated with dislocation of the proximal radioulnar joint.^1^ It is relatively rare and affects 2 to 5% of the population. ^(^ ^2^

Bado was the first to classify this injury into four types. He observed that in all types, the ulnar fracture and the dislocation of the radial head presented the same direction, except for Type IV, in which the radial fracture is located at the same level of the ulna. ^(^ ^1^ Type II injuries, although theoretically associated with lower-energy trauma, can still lead to complications. The resulting chondral injury from joint fractures and associated ligament injuries can cause radiological complications, leading to heterotopic ossification, osteolysis around the prosthesis, and loosening of the prosthesis, along with pseudarthrosis as well as functional complications, such as instability, pain, and restricted elbow mobility. ^(^ ^3^

Jesse Jupiter observed that Bado Type II fractures (posterolateral dislocation of the radial head) could be associated with fractures at the proximal ulna, and he divided them into four types: Type IIA, which affects the proximal olecranon and the coronoid process; Type IIB, which affects the metaphyseal-diaphyseal junction and does not involve the coronoid process; Type IIC, which affects the ulnar shaft diaphysis; and Type IID, which affects the entire proximal ulna and involves the coronoid process. Every subtypes of the Jupiter classification are associated with the radial head fracture. ^(^ ^1^ ^),(^ ^4^

Jupiter’s Monteggia Type II fractures present complex surgical treatment that mainly aims to restore the ulna length and anatomic reduction of the joint. Radial head fractures can be treated with osteosynthesis, resection, or arthroplasty. Also, ligament injuries should be identified and treated. Similarly, in cases of coronoid fracture, it must be repaired for better joint stability. ^(^ ^5^ ^),(^ ^6^ Despite the adequate treatment of all lesions, postoperative complications are frequent. ^(^ ^7^

There are few studies that exclusively evaluate the complications of Jupiter’s Monteggia Type II fractures. Most articles bring a miscellany of complex elbow injuries such as Hotchkiss’ terrible triad, Monteggia, and transolecranial fractures. ^(^ ^2^ ^),(^ ^3^ ^),(^ ^5^ ^),(^ ^8^ ^)-(^ ^11^

### Objectives

This study main objective was to evaluate the incidence of postoperative and secondary radiographic and functional complications. Additionally, it aims to correlate possible risk factors that may influence the functional outcome of the elbow after surgical treatment of Jupiter’s Monteggia Type II fractures.

## METHODS

This is a retrospective study that reviews the medical records of participants who underwent surgery for correction of Jupiter’s Monteggia Type II fractures at a Reference Hospital from 2019 to 2021.

The inclusion criteria were:

- surgically treated Monteggia Type II fractures according to Jupiter’s classification
- a minimum follow-up time of one year after surgery
- have pre- and postoperative radiograph images in anteroposterior and lateral views for classification of fractures and evaluation of complications
- have preoperative tomography for classification of fractures

The exclusion criteria were:

- patients who did not sign an informed consent form
- patients who did not have complete information in their medical records
- patients who had associated fractures in the ipsilateral limb or polytraumatized patients.

All participants signed an informed consent form.

The Mayo Elbow Performance Score (MEPS) was applied to assess postoperative elbow function. Moreover, demographic data were evaluated, including gender and age, along with the affected side and the specific subtype of Monteggia Type II fractures according to Jupiter’s classification.

Orthopedic physicians, who did not participate in the surgical treatment, conducted the radiological evaluation using the Synapse^®^ program. Consolidation was considered complete when the fracture line disappeared completely (primary consolidation) or when complete cortical bridging in three cortices were found (when primary consolidation was not achieved). Also, the formation of heterotopic ossification, ^(^ ^7^ the presence of radial head subluxations or radial head prosthesis, the presence of osteolysis and/or radiographic loosening around the radial head prosthesis were evaluated. Osteolysis was considered when radiological examination identified signs of radiolucency around the prosthesis stem. ^(^ ^2^

Surgeries were performed with the patients in lateral decubitus position, using the global posterior approach to expose the elbow. In cases of comminuted ulnar fractures (Jupiter Type IID), the treatment procedure initially addressed radial head fracture via osteosynthesis using a 2.7 mm plate and/or Herbert screw. Arthroplasty (Metabio^®^ prosthesis) was used as an alternative approach. Ulnar fractures were treated with a 3.5 mm reconstruction plate, and the coronoid fractures were treated with traction screws or support plates. Small coronoid fragments were fixed with transosseous sutures. After approaching the ulna and the radial head, joint stability was tested and, if there were signs of lateral or medial instability, the repair was performed with a 3.5 mm anchor.

The Monteggia Type II fractures according to Jupiter and the incidence of complications were compared by the Chi-square test or Fisher’s exact test. Non-categorical variables were tested by the Kolmogorov-Smirnov test. Thus, in the study of these variables, both unpaired *t*-test (parametric variables) and Pearson’s test were used. All analyses were conducted using PASW Statistics 28.0 program (SPSS Inc., Chicago, USA), adopting a 5% significance level (p < 0.05).

The study was approved by the local Research Ethics Committee under number 58417322.9.0000.5404

## RESULTS

A total of 15 patients were evaluated after inclusion criteria. The mean age was 53 ± 15 years. The youngest patient was 29 years old and the oldest was 80 years old.

Patients’ mean follow-up time was at least one year after surgery (23.2 ± 8.9 months). Most participants were men (twice higher than women) and the left side was the most affected.

**Figure 1:**
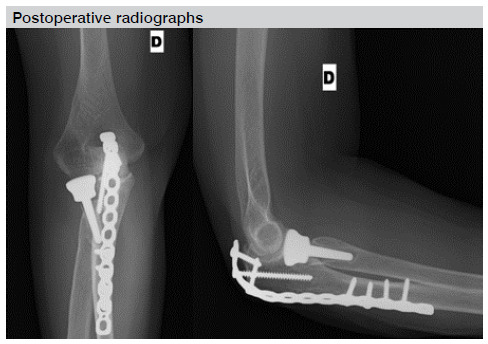
Osteolysis around the radial head prosthesis as a complication. Postoperative radiographs

Most patients who underwent radial head replacement developed signs of osteolysis around the prosthesis but none of the arthroplasties showed migration or loosening. However, all patients remained asymptomatic and, therefore, were not surgically reapproached (Figure 1).

**Figure 2:**
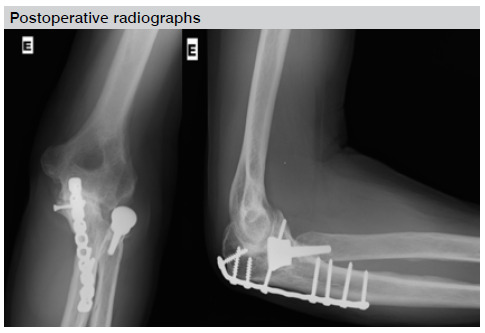
Posterolateral rotatory instability of the elbow as a complication. Postoperative radiographs

The main postoperative instability occurred in the lateral collateral ligament with evolution to posterolateral rotatory instability. One case was subjected to lateral ligament reconstruction using the ipsilateral palmaris longus tendon and two cases were subjected to prosthesis removal since it presented, in addition to instability, signs of component loosening (Figure 2).

The most prevalent fracture was Jupiter Type IIA (seven cases) followed by IID (five cases) (Table 1).

**Table 1:** Description of demographic data.

Characteristic	Value
Age [mean (± SD)] (years)	53.15 ± 15.0
**Gender [No. (%)]**	
Men	10 (66.7)
Women	5 (33.3)
**Jupiter classification [No. (%)]**	
IIA	7 (46.7)
IIB	2 (13.3)
IIC	1 (6.7)
IID	5 (33.3)
**Affected side [No. (%)]**	
Right	5 (33.3)
Left	10 (66.7)

The most frequent complications were heterotopic ossification and osteolysis around the radial head prosthesis (Table 2).

**Table 2:** Incidence of postoperative complications.

Complication	Value
Heterotopic ossification (n/%)	7 (46.7)
Osteolysis around prosthesis (n/%)	7 (80)
Elbow instability	3 (20)
Pseudarthrosis	1 (6.6)

N: number; %: percentage.

The only case of pseudarthrosis evolved asymptomatically, requiring no surgical treatment.

According to the MEPS functional score: six patients had excellent results, one patient had good results, two patients had fair results, and six patients had poor results. Thus, 53% of patients evolved to an unfavorable functional outcome. Table 3 illustrates the patients’ functional outcomes (Table 3).

**Table 3:** Patients’ functional outcomes - Mayo Elbow Performance Score (MEPS) distribution per patient and total values (mean ± SD).

	Patient	Pain (points)	ROM (points)	Stability (points)	Daily Function (points)	Total (points)
	1	45	20	10	25	100
	2	45	15	10	0	70
	3	15	15	5	0	35
	4	15	15	10	20	60
	5	45	20	10	25	100
	6	45	20	10	25	100
	7	45	20	10	25	100
	8	30	20	10	20	80
	9	15	15	5	0	35
	10	45	20	10	25	100
	11	45	20	0	25	90
	12	15	5	0	0	20
	13	15	15	10	0	40
	14	0	5	0	10	15
	15	0	5	5	0	10
Total (mean ± SD)	15	28 ± 17.2	15.3 ± 5.6	7 ± 4	13.3 ± 11.4	63.6 ± 33.6

SD: standard deviation; ROM: range of motion.

The presence of the coronoid fracture (p = 0.005) and the Jupiter Type IID fracture (p = 0.006) evolved with functional worsening (MEPS score).

Radial head replacement did not evolve with functional difference when compared to osteosynthesis.

Furthermore, the progression to pseudarthrosis, presence of osteolysis, whether or not associated with prosthesis loosening, and the presence of heterotopic ossification did not influence the functional outcome (Table 4).

**Table 4:** Correlation between the various factors and MEPS - association of MEPS with complications.

	Values according to MEPS Score (Mean ± SD)			
Characteristic	Presence	Absence	CI	P-value^a^
Osteolysis (Prosthesis)	72.14 ± 37.62	60.00 ± 34.46	-59.38-35.10	0.28
Coronoid fracture	56.25 ± 34.91	93.3 ± 11.54	10.5-63.6	0.005
Loosening (prosthesis)	50.00 ± 36.05	67.08 ± 35.25	-32.25-66.42	0.74
Jupiter Type IID	35.00 ± 22.91	78 ± 31.10	8.87-77.12	0.006
Heterotopic ossification	61.43 ± 38.0	65.63 ± 34.27	-36.68-45.07	0.41

^a^ T-test; CI: confidence interval.

## DISCUSSION

Jupiter’s Monteggia Type II fractures are rare and can affect older patients with low bone density as a result of low-energy trauma. ^(^ ^8^ However, such injuries can also occur due to high-energy trauma, especially with direct impact to the anterior or posterior elbow, such as a direct blow. ^(^ ^6^ In our study, the highest prevalence was observed in young men (66.7%), which differs from some European studies that reported a higher prevalence in women. ^(^ ^2^ ^),(^ ^3^ This difference may be related to the higher incidence of traffic accidents in developing countries like Brazil. ^(^ ^9^ High-energy traumas result in more significant chondral injuries and may have a greater potential for complications. ^(^ ^4^

Jupiter Type IIA (46.7%) and Type IID (33.3%) were the most prevalent fractures in our study. Laun et al. ^(^ ^2^ and Calderazzi et al. ^(^ ^3^ observed a higher prevalence of Type IIB, probably reflecting a demographic profile of older patients with lower-energy fractures when compared to our results. ^(^ ^10^

The sum of Type IIA and IID lesions found in our results represents 80% of the cases. Therefore, only 20% of patients did not have a fracture of the coronoid process associated with the presence of the coronoid fracture. The coronoid process, which is an important restrictor of the elbow, can generate joint instability when fractured. The literature diverges in whether the presence of the coronoid fracture may be a factor for worse prognosis. Our results corroborate a study by Suarez, Barquet, and Fresco, ^(^ ^12^ who observed worse functional outcomes. ^(^ ^4^ ^),(^ ^13^ However, in the study by Chemama et al. ^(^ ^14^ in 2010, better MEPS values were observed for patients who underwent fixation of the coronoid process compared to those who were not fixed, but the authors did not perform statistical analysis of their results. ^(^ ^15^

Heterotopic ossification is a common complication in joint fractures, especially around the elbow. ^(^ ^7^ ^),(^ ^12^ Although ossification occurred in 46.7% of cases, this complication did not result in functional worsening, probably because it did not lead to a decrease in the elbow range of motion. Egol et al. ^(^ ^16^ described the development of heterotopic ossification in 22% of the patients studied and did not correlate ossification with functional worsening.

Most patients underwent radial head arthroplasty. Radial head fractures located in the articular surface of the proximal radioulnar joint or involving more than three fragments are difficult to treat with rigid osteosynthesis that allows early mobility, thus requiring prosthetic replacement. ^(^ ^17^ According to MEPS, no disparities were found between patients who underwent arthroplasty and osteosynthesis. However, the literature presents no conclusion regarding the best approach to elbow fractures/dislocations associated with radial head fractures. ^(^ ^6^ ^),(^ ^11^ ^),(^ ^16^ ^),(^ ^17^ Konrad et al. ^(^ ^6^ and Egol et al. ^(^ ^16^ reviewed cases similar to ours and observed no differences in functional scores regardless of how the radial head replacement was conducted, corroborating our outcomes. However, Ring, Jupiter, and Simpson^18^ identified 26 patients with Bado Type II fractures associated with radial head fracture. There were seven Mason II and 19 Mason III fractures, and the cases were treated with different approaches, that is, from conservative management to radial head excision, open reduction internal fixation (ORIF), and prosthetic replacement. However, the authors noted that all patients obtained unsatisfactory results. ^(^ ^11^ Similarly, Matar et al., ^(^ ^17^ in their study series with 18 patients, concluded that the postoperative functional outcome of their patients did not depend on the severity of the fracture, but rather on how the fracture was surgically treated.

Osteolysis around the stem of the radial head prosthesis occurred in 80% of the patients, with 42% experiencing migration (loosening). However, it was necessary to remove or revise the component in no patient. The presence of osteolysis or prosthesis loosening was not correlated with a functional worsening. Some articles agree that radiological signs of loosening may evolve without functional changes because mobility, especially in pronation-supination, occurs in the stem of the prosthesis, which has a minimal impact on the range of motion of the elbow. ^(^ ^2^

Only one case evolved to ulnar pseudarthrosis, which was asymptomatic. Thus, there was no need for a additional surgical procedures. Probably, since there was no associated plate breakage, sufficient stability was achieved at the fracture site, resulting in minimal local pain. High-energy fractures, especially open fractures, can cause bone devascularization, increasing the likelihood of progressing to pseudarthrosis. ^(^ ^19^

The case of posterolateral instability, which did not present clinical loosening of the radial head prosthesis, underwent lateral ligament reconstruction using an ipsilateral palmaris longus tendon graft. However, there was no functional discrepancy observed between the use of the prosthesis, ORIF, or resection in the studied patients. Laun et al., ^(^ ^2^ did not observe any case of postoperative instability. On the other hand, Ring, Jupiter, and Simpson^18^ observed that several complications required early reoperation in nine of their patients and, in one of these cases, there was persistent ulnohumeral instability. They believed, retrospectively, that this instability was due to posterolateral rotatory instability resulting from damage to the lateral collateral ligament caused by posterior displacement of the radial head and residual malalignment of the coronoid process. ^(^ ^18^

According to the MEPS functional score, 53% of the patients evolved with unfavorable outcomes. The mean score was 63.3 points. However, radial head replacement did not evolve with functional difference when compared to osteosynthesis. With a score value closer to that found in our study, Matar et al. ^(^ ^17^ obtained a mean MEPS score of 76.6 points, also not showing statistical difference in the way that the radial head fracture was managed. Giannicola et al. ^(^ ^5^ obtained a score of 98 points; however, their study encompasses a range of complex elbow injuries, not limited to Jupiter’s Monteggia Type II fractures.

The presence of the coronoid fracture (p = 0.005) and the Jupiter Type IID fracture evolved with functional worsening (MEPS score). Egol et al. ^(^ ^16^ mentioned that Jupiter’s Type IID fractures had a higher chance of pseudarthrosis and need for a new surgical approach with worse functional outcomes. Josten and Freitag, ^(^ ^20^ observed that patients with Type IIA fractures evolved with decreased elbow range of motion and required additional surgical procedures. Konrad et al. ^(^ ^6^ observed that Types B and C fractures usually evolve with good or excellent results, whereas Types A and D fractures presented worse functional outcomes. Furthermore, Type IIA fractures have the worst long-term functional evolution.

Our study has some limitations such as the retrospective design and small sample size. However, most studies on this topic include various types of complex elbow fractures, rather than specifically focusing on Monteggia Type II fractures according to Jupiter’s classification.

## CONCLUSION

Jupiter’s Monteggia Type II fractures evolved with high rates of postoperative complications. The main complications were elbow functional worsening and osteolysis around the radial head prosthesis. Jupiter’s Type IID fractures and associated coronoid fractures evolved with worse functional outcomes. The main reason for reoperation was posterolateral rotatory instability of the elbow.
